# Experimental Comparison of Photothermal Conversion Efficiency of Gold Nanotriangle and Nanorod in Laser Induced Thermal Therapy

**DOI:** 10.3390/nano7120416

**Published:** 2017-11-26

**Authors:** Yatao Ren, Qin Chen, Hong Qi, Liming Ruan

**Affiliations:** School of Energy Science and Engineering, Harbin Institute of Technology, Harbin, 150001, China; renyt@hit.edu.cn (Y.T.R.); chenqinhit@163.com (Q.C.)

**Keywords:** gold nanoparticle, photothermal conversion, nanotriangle, nanorod, photothermal therapy

## Abstract

An experimental comparison of the photothermal conversion efficiency (PCE) for gold nanotriangles (GNTs) and nanorods (GNRs) was carried out in the present work. The discrete dipole approximation method was applied to identify the spectral characteristic of GNTs and GNRs with different aspect ratios. On this basis, the PCE of GNTs and GNRs in photothermal therapy were compared theoretically. Afterwards, an in vitro experiment was adopted to investigate the thermal effect of porcine muscle induced by laser irradiation, with and without injected GNTs and GNRs. The influences of laser total power, nanoparticle concentration, and nanoparticle type were investigated. It was found that for the commonly-used wavelengths for photothermal therapy, the PCE of GNTs is higher than that of the GNRs. Furthermore, for GNRs loaded in tissue in vitro, high laser power and high concentration of nanoparticles leads to the degeneration and even carbonization of tissue. However, for the GNTs with the same situation (laser power, nanoparticle volume concentration, and heating time), it could lead to the tissue’s evaporation instead of carbonization.

## 1. Introduction

Thermal therapy is a newly developed promising alternative treatment of conventional surgery and chemoradiotherapy for benign disease or cancer [1,2,3,4,5]. According to the different techniques applied to induce hyperthermia of biological tissue, it can be divided into a few categories, such as radiofrequency [6], ultrasonic [7], microwave [8], magnetism [9], and laser induced thermal therapy (LITT) [10], to name a few. Due to their minimally invasive or even non-invasive nature, these techniques are especially applicable for patients who are not suitable for radiotherapy, chemotherapy, or surgical therapy. However, they suffer from the drawback that nonselective heating can damage nearby healthy tissue [10,11,12]. Therefore, specific thermal conversion agents, such as thermoseeds and nanoparticles, are introduced to the process of thermal therapy to improve the heat generation in cancerous cells and tissues. Among these thermal therapy techniques, the LITT is a promising focal therapy against tumors since the irradiating laser can be precisely controlled. In the LITT, strong optical absorption and tunable resonance wavelength allows the utilization of metal nanoparticles [13,14,15,16,17,18,19,20,21,22], among which, the gold nanoparticles, such as gold nanorods (GNRs), nanoshperes, and nanoshells are commonly employed as photothermal conversion agents for LITT due to their superior optical, chemical, and biological properties [16,20,23,24]. Meanwhile, the laser wavelength used in LITT is always chosen from the “optical window” of biological tissue (600–1300 nm) [25] to reduce the absorption of healthy tissue and, therefore, to minimize the unwanted temperature rise.

The morphology of nanoparticles plays an important role in the application of LITT since nanoparticles with different shapes will have different surface plasmon resonance characteristics. To date, the optimal shapes of gold nanoparticles for photothermal therapy has been the research interest for scholars all over the world [12,13]. The application in thermal therapy requires that nanoparticle candidates have a tunable absorption peak (favorably in the near-infrared region) and a high photothermal conversion efficiency (PCE), which makes nanosphere an inadequate choice since its absorption peak is in the range of the visible spectrum [18]. The gold nanorods are thought to be one of the most effective photothermal conversion agents, owing to their high tunable aspect ratio and strong biocompatibility [14,26]. A large amount of research has been conducted to investigate the performance of GNRs in the application of photothermal therapy [27,28]. Meanwhile, the integration of cancer diagnosis and treatment puts forward higher demands for nanoparticle properties. In recent years, a number of different shapes of nanoparticles are developed as photothermal conversion agents, including nanotriangles [29,30,31], nanostars [32], nanocages [33,34], nanoflowers [35], etc. Outstanding optical properties of gold nanotriangles (GNTs) are illustrated owing to its high aspect ratio [36], which makes GNTs a promising candidate for nanomedicine and other related areas.

To test the performance of nanotriangles, researchers have focused their efforts in both the therapeutic and diagnostic aspects. Most recently, in the application of LITT, Luo and co-workers [37] applied the silver nanotriangles for uveal melanoma therapy. They found that the silver nanotriangles exhibited good biocompatibility and stability. Also, significant cell death was observed in the in vivo and in vitro treated cancer cells. For the application of GNTs as both contrast and thermal conversion agents, Bao and co-workers [38] developed a hybrid approach for both imaging and focal thermal therapy with the assistance of GNTs. It was proved to be highly efficient for imaging with high-resolution blood vessels and enhanced localized hyperthermia. The GNTs show great potential in the application of nanoparticle assisted cancer diagnosis and therapy. However, to the best of our knowledge, there is limited research concerning the comparison of photothermal conversion efficiency of GNTs and GNRs in the application of LITT. Therefore, in the present work, the PCE of GNTs and GNRs in photothermal therapy were compared experimentally.

To evaluate the optical properties of different kinds of nanoparticles, both theoretical and experimental tools should be applied. For theoretical solutions, the Maxwell’s equations needs to be solved to obtain the spectral optical properties of small particles. The analytical solution can be obtained by Mie theory [39]. However, it is only suitable for homogeneous and isotropic spheres or spheroids and core-shell nanospheres. The discrete dipole approximation (DDA), which is one of the most versatile, important, and widespread numerical methods to obtain the optical properties of small particles with arbitrary shapes and compositions, has been extended to the application of near-field recently [40]. The in vivo or in vitro experiment also need to be executed to test the performance of gold nanoparticles. A high photothermal conversion efficiency is regarded as one standard for the judgment of applicability for nanoparticles in LITT. In the present work, an experimental and theoretical comparison of photothermal conversion efficiency of GNTs and GNRs are presented. The DDA package was employed to study the spectral optical properties of GNRs and GNTs. On this basis, the experiments for the thermal effects of the laser and in vitro tissue interaction were carried out. Also, simulated results by the COMSOL model were established to compare with the experimental results.

## 2. Results

### 2.1. Spectral Characteristics of Gold Nanotriangles (GNTs) and Nanorods (GNRs)

Through the whole paper, if not otherwise specified, for GNRs, the direction of the electric field is parallel to the long axis, and for GNTs, the direction of the electric field is parallel to the prism cross section. The dielectric constants of gold nanoparticles are regarded as the same as bulk metal. The data was obtained from Ref. [41]. The refractive index of the surrounding media is assumed to be 1.33, which is similar to pure water.

In this section, the PCE of GNTs and GNRs are compared theoretically. The experimental measurement of PCE in biological tissue is a complicated problem that needs to be further investigated. The spectral optical properties of GNTs and GNRs were calculated by using the DDA method. The absorption quantum yield *η* can be applied as one of the evaluation indices of PCE, which can be expressed as:

(1) $$\eta =\frac{Q_{\mathrm{abs}}}{Q_{\mathrm{ext}}}$$

where *Q*_abs_ and *Q*_ext_ are the absorption and extinction efficiency of nanoparticles, respectively.

**Figure 1:**
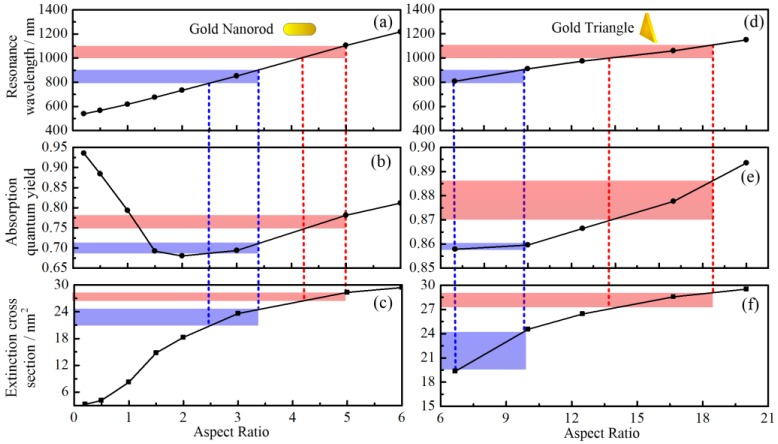
Correlation between resonance wavelength, absorption quantum yield, and extinction cross section with the aspect ratio, respectively. (**a**–**c**) nanorod; (**d**–**f**) nanotriangle. The color bars indicate the results corresponding to commonly used laser wavelengthes.

Figure 1 shows the resonance wavelength, absorption quantum yield, and extinction cross section of GNTs and GNRs in different aspect ratios with effective radius *R*_eff_ = 20.2 nm. The aspect ratio of GNRs is the ratio of particle length (not including hemispherical end-caps) and cylinder diameter. For GNTs, it is the ratio of triangle edge length and particle thickness.

**Figure 2:**
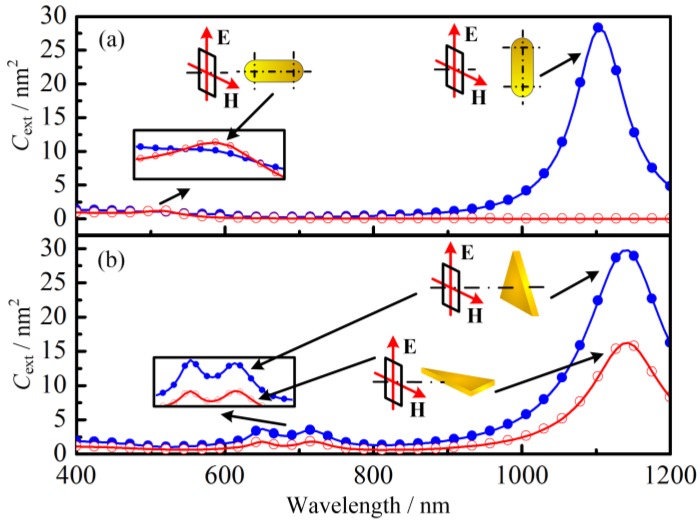
Extinction cross section of (**a**) gold nanotriangles (GNTs) and (**b**) gold nanorods (GNRs) with different incident direction of electromagnetic waves. The results are the average value of extinction cross section when the incident electric field direction is horizontal and vertical. The insets show the partial enlarged views of local maximum. E and H stands for electric and magnetic fields, respectively.

The above results are based on the condition that the incident electromagnetic wave is fixed at a certain direction. However, in the actual application, the nanoparticles can be irradiated by the incident electromagnetic wave in any direction, which may influence their absorption properties significantly. Therefore, the spectral optical properties of GNTs and GNRs irradiated by electromagnetic wave from different directions were investigated in the present research (see Figure 2). It is interesting to note that for the GNTs, the spectral extinction cross sections have the same trend even when the incident electromagnetic wave is from different directions. However, for GNRs, the resonance wavelength is highly related to the incident direction. In another word, the optical properties of GNTs are less sensitive to their own orientations. The optical properties can decrease from their maximum to almost zero with the change of orientation. This means in gold nanoparticle solutions, since the nanoparticles are randomly oriented, the average absorption cross section of GNR will be smaller than that of GNT.

### 2.2. Thermal Response of Tissue In Vitro Embedded with Nanoparticles

#### 2.2.1. Laser Radius Measurement

**Figure 3:**
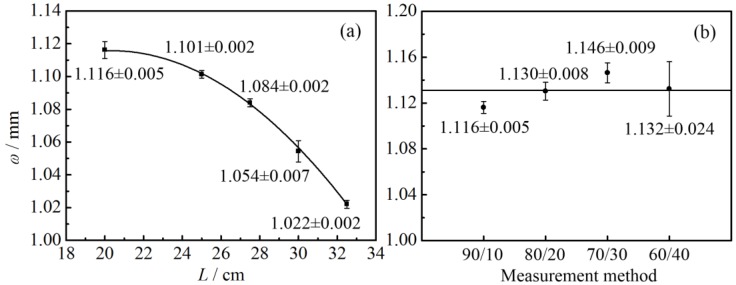
Measurement results of laser radius. (**a**) Laser radius in different locations; (**b**) laser radius measured by different methods.

The laser energy profile was measured first. The measurement results are illustrated in Table 1 and Figure 3. *L* stands for the distance between the measurement location and laser outlet. It can be seen that within the measurement range under consideration, the laser radius decreases with the increasing of *L*. The radius of the laser was around 1 mm. Furthermore, the standard deviation of the measured laser radius using different methods (90/10, 80/20, 70/30, and 60/40 method) presents an upward tendency. This is because for the laser beam with Gaussian distribution, the energy density and its gradient are higher when it is close to the center of the laser beam. Therefore, the slight movement of the knife-edge could cause a large variation of the energy received by the detector, which may lead to a relatively large error in the measurement of the knife position. In conclusion, the 90/10 method is recommended for the measurement of laser radius. 

#### 2.2.2. Laser Heating Effect

**Figure 4:**
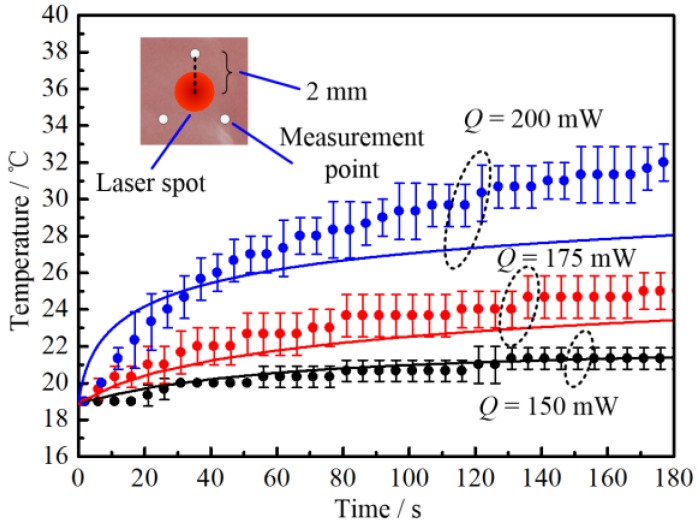
Temperature response of in vitro biological tissue under different laser intensities. Solid lines are simulated results. Scattered circles with error are experimental results. The inset indicates the laser irradiated spot (red circle) and measurement positions (white circles).

The temperature rise of porcine muscle without nanoparticles was measured under different laser intensities (see Figure 4). *Q* stands for the laser total power. The room temperature was 19 ± 1 °C. To reduce the influence of thermocouple on the projected laser beam, the temperature measurement points were arranged far from the laser beam center. From the above measurements, it can be seen that the radius of the laser was approximately 1 mm. Therefore, the thermocouple was positioned as shown in the inset of Figure 4. It can be seen that the temperature rise of tissue increases with the increase of laser power. Also, when the laser total power is smaller than 150 mW, there is no apparent temperature change. The COMSOL model was also applied to simulate the temperature change in tissue in vitro. The simulated results are shown as a solid line in Figure 4. The light propagation in participating media, like tissue, can be calculated by different methods [42,43]. In the present work, the Monte Carlo (MC) method was adopted. The details of the COMSOL model and the MC method are available in our former work [2], which will not be repeated here.

**Figure 5:**
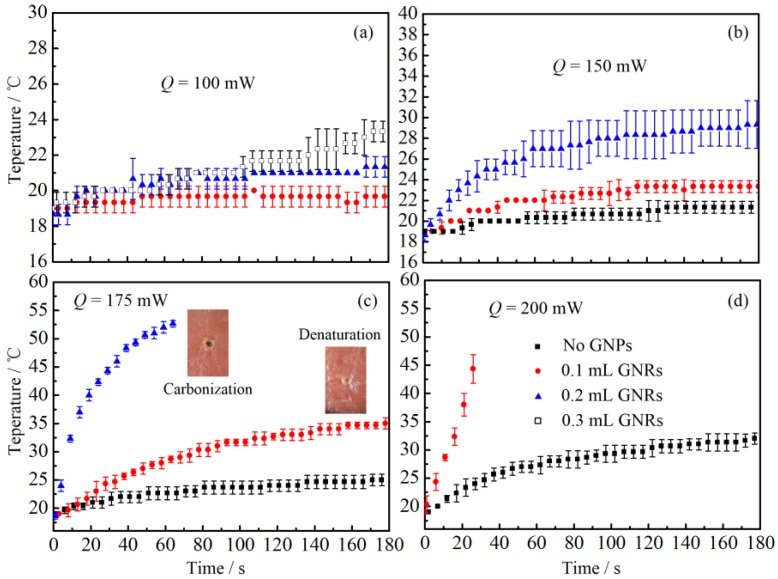
Temperature changes of biological tissue after injecting different amount of GNR solution: (**a**) *Q* = 100 mW; (**b**) *Q* = 150 mW; (**c**) *Q* = 175 mW; (**d**) *Q* = 200 mW. *Q* stands for the laser total power.

**Figure 6:**
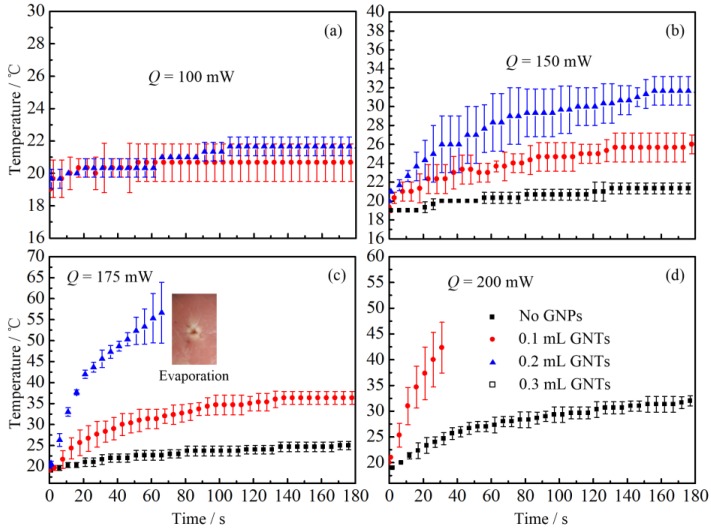
Temperature changes of biological tissue after injecting different amount of GNT solution: (**a**) *Q* = 100 mW; (**b**) *Q* = 150 mW; (**c**) *Q* = 175 mW; (**d**) *Q* = 200 mW.

Afterwards, to investigate the PCE of gold neanorod and nantriangle, we studied the temperature change of porcine muscles injected with different kinds and amounts of nanoparticles, as illustrated in Figure 5 and Figure 6.

**Figure 7:**
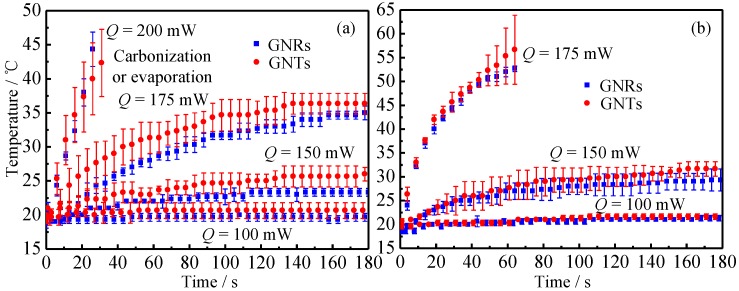
The impact of different kinds of gold nanoparticles, nanorods and nanotriangles, under different injection dosage: (**a**) 0.1 mL and (**b**) 0.2 mL.

Figure 7 shows the comparison of temperature change of tissue injected with GNRs and GNTs. The mass concentration of GNR and GNT solutions are the same. Therefore, under the same injection dose, the total volume of GNRs and GNTs inside the tissue should be the same.

## 3. Discussion

### 3.1. Spectral Characteristic of GNRs and GNTs

As shown in Figure 1, the resonance wavelength of GNRs and GNTs increases with increasing aspect ratio. In the present work, we focused on the application of gold nanoparticle in laser induced photothermal therapy. In Figure 1, two commonly-used wavelengths for photothermal therapy (850 and 1064 nm) are indicated. It can be seen from Figure 1c,f that the extinction cross section for GNRs and GNTs are almost the same (about 28 nm^2^ for 1064 nm and 22 nm^2^ for 850 nm). However, the absorption quantum yield of GNTs is higher than that of GNRs at the same wavelength. For example, at 1064 nm, the absorption quantum yield of GNTs is between 0.87 and 0.885; for GNRs, it is between 0.75 and 0.78. The interaction between gold nanoparticles and incident electromagnetic wave eventually decides the absorption and scattering cross section of different shapes of gold nanoparticles. For GNTs, it has a larger absorption cross section when it has the same scattering cross section with GNRs, which is to say, it has a larger PCE. To be specific, when the GNTs and GNRs are irradiated by the same amount of laser, the GNTs will absorb more light and convert it into heat, which makes it an outstanding candidate for LITT.

From Figure 2, it is interesting to note that for the GNTs, the spectral extinction cross sections have the same trend even when the incident electromagnetic wave is from different directions. However, for GNRs, the resonance wavelength is highly related to the incident direction. In other words, the optical properties of GNTs are less sensitive to their own orientations. The properties can decrease from their maximum to almost zero with the change of GNT orientation, which is also found in other references [44]. In the thermal therapy, the extinction cross section will be the average for every direction. This means, even though the cross sections for GNTs and GNRs are the same for the situation in Figure 1c,f, the average cross section of GNRs will still be smaller than that of GNTs at the resonance wavelengths. From the analysis above, it can be concluded that the absorption quantum yield and extinction cross section of GNTs are both larger than those of GNRs if they have the same effective radius. Therefore, the photothermal conversion efficiency of GNTs is higher than that of GNRs generally.

### 3.2. Thermal Response of Tissue In Vitro Embedded with Nanoparticles

From Figure 4, it can be found that when the laser total power is relatively low (*Q* = 150 mW), the simulated results match well with experimental results. However, when the laser power increases (*Q* = 175 and 200 mW), the simulated temperature becomes lower than the experimental one. This is because high temperature leads to water loss of in vitro tissue due to evaporation, which will cause the increase of thermal conductivity and decrease of heat capacity. It is interesting to note that when laser power reaches 200 mW, the simulated temperature is higher than the experimental one in the early stage. This is basically due to two reasons: (1) when the heating rate is high, there is a hysteresis phenomenon in temperature measurement of the thermocouple; (2) there is tremendous heat loss associated with the water evaporation, which is not taken into consideration in the heat transfer model. Therefore, the numerical model is only suitable for low-temperature situations and still needs to be further improved by taking into consideration water evaporation, which will be also be the focus of our further investigations.

From Figure 5 and Figure 6, it can be seen that there is a significant increase of temperature when tissue is injected with gold nanoparticles. When the laser total power is 100 mW, no temperature change is observed for tissue without nanoparticles. However, there is still an apparent temperature rise even when only a small dose of GNPs is injected (see Figure 5a and Figure 6a). Furthermore, when the laser power is larger than 175 mW, the tissue with GNPs may suffer from denaturation or even carbonization and evaporation (see Figure 5c). Meanwhile, when no GNPs are injected, no visual morphologic change can be observed in the porcine muscle. It should be noted that due to the injection of GNP solution, the water content of tissue increases. Therefore, the temperature of tissue should decrease due to the evaporation related heat loss, which means the real temperature rise caused by the photothermal conversion of GNPs should be much higher excluding the water evaporation effect. It is worth noting that the tissue injected with nanorod solution may suffer from carbonization when the laser power is too high (see the insect of Figure 5c). However, for tissue injected with nanotriangles, the tissue under laser irradiation may be evaporated instead of carbonized (see the insect of Figure 6c). Tissue carbonization and evaporation depend on the energy accumulation and diffusion inside tissue. The tissue evaporation or carbonization may occur when the temperature of biological tissue is higher than 100 °C [45]. However, the evaporation of tissue is caused by the local explosion induced by the rapid expanding of water evaporation [45]. It also can be called thermomechanical effect or photoablation, which is a much more severe process. Therefore, we think the evaporation is induced by a rapid temperature rise.

It can be seen from Figure 7 that before the tissue’s carbonization and evaporation, the temperature rise caused by GNTs are higher than that of the GNRs. From the analysis of Section 3.1, it can be concluded that the PCE of GNTs is higher than that of GNRs. In other words, if the same amount of light projects to the nanoparticles, the GNTs can absorb more optical energy and convert it into thermal energy, compared to GNRs. When in vitro tissue embedded with gold nanoparticles is irradiated by laser, the tissue with GNTs will absorb more light and, therefore, has a higher temperature rise than that with GNRs. In gold nanoparticle assisted LITT, nanoparticles (GNRs or GNTs) with specific surface modifications will accumulate inside the tumor. When the tumor is irradiated by laser, the temperature of the tumor will rise due to the absorption of gold nanoparticles. Then, the cancer cell will be damaged by hyperthermia. Since GNTs can lead to a higher temperature rise than GNRs, they can be regarded as a better photothermal conversion agent.

## 4. Materials and Methods 

### 4.1. Gold Nanoparticles

**Figure 8:**
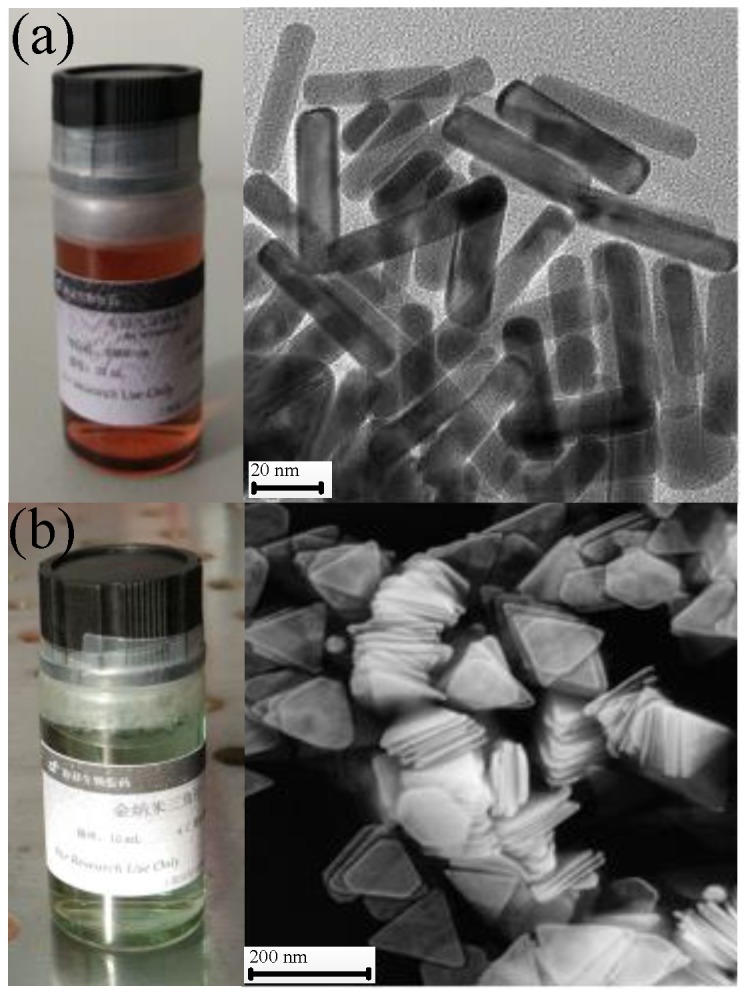
SEM image of GNR and GNT solutions. (**a**) GNRs; (**b**) GNTs.

The GNT and GNR solutions were purchased from the Shanghai So-Fe Biomedicine Co., LTD. (Shanghai, China), the mass concentration of which was 0.1 mg/mL for both. The aspect ratio of GNRs is 6.5:1. There are two absorption peaks in the UV-Vis spectra (Beijing, China) of a GNR solution, which are 510 and 1060 nm. For the GNTs, the side length of triangular cross section is 140 ± 25 nm. The thickness of GNTs is 8 ± 2 nm. There are three absorption peaks in the UV-Vis spectra of GNT solution, which are 401, 500, and 1060 nm. Figure 8 shows the scanning electron microscope (SEM) image of GNR and GNT solutions.

### 4.2. The Discrete Dipole Approximation (DDA)

The DDA method can be applied to calculate the optical properties of particles with arbitrary geometries and compositions. The DDA package, developed by Draine and Flatau [46], has been widely employed. The basic principle of DDA is to discretize a small particle into a cubic array of virtual *N*-point dipoles [47]. Then, the scattering field of the whole particle can be approximated as the summation of all the dipoles. Therefore, the DDA can be employed to calculate the optical properties of arbitrary shape targets theoretically if it is properly discretized. The detailed description and mathematical formulation of DDA can be found elsewhere [31,46,48,49]. For the sake of simplicity, it will not be repeated in the present work.

### 4.3. Knife-Edge Method

To accurately predict the thermal response of the interaction between laser and biological tissue, the laser beam radius and spatial energy profile should be measured first. The radius can be measured by slit scan method, pinhole method, charge-coupled device (CCD) method [50,51], etc.. The knife-edge method is often employed as a standard method for Gaussian laser beam characterization, since it is a beam profiling method that allows quick, inexpensive, and accurate identification of beam parameters [50,52,53]. Therefore, in the present work, the knife-edge method was applied. The schematic diagram of the knife-edge method is illustrated in Figure 1. The Gaussian laser beam profile can be expressed as follows [52]:

(2) $$I\left(r,z\right)=I_{0}\left(z\right)\exp\left[-2r^{2}/\omega^{2}\left(z\right)\right]$$

where *I*_0_ is the power intensity in the center of the laser beam, and *ω*(*z*) is laser beam radius in position *z*. The definition of the laser radius in the present work is shown in Figure 1b, which is the length between the laser center and the position where the laser power *I* [*ω*(*z*), *z*] equals *I*_0_/*e*^2^.

The procedure of measuring the laser beam diameter is presented below.

**Figure 9:**
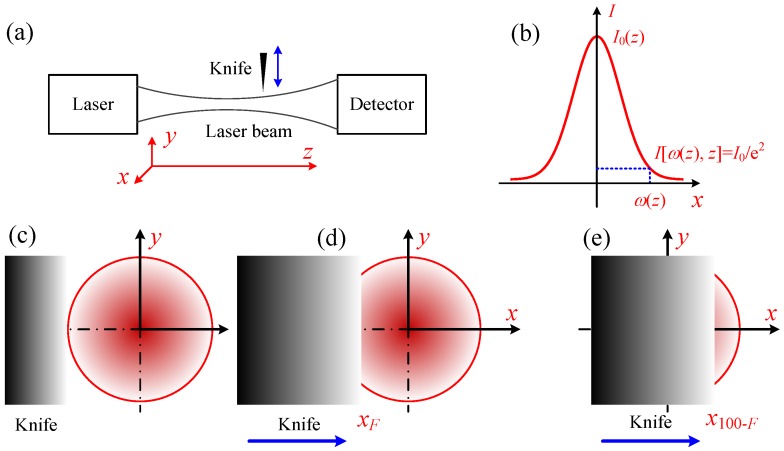
Principle of knife-edge method. (**a**) Schematic of measuring laser intensity distribution using knife-edge method; (**b**) definition of laser radius; (**c**–**e**) procedure of knife-edge method.

Step 1. The photoelectric detector was applied to measure the total intensity of the laser beam, as shown in Figure 9a,c;

Step 2. The knife was moved slowly along the radial direction of the laser beam while monitoring the intensity of the laser at the same time. When the total energy received by the detector reached *F*% (shown in Table 2) of the total laser intensity, we recorded the position of the knife as *x_F_*, as shown in Figure 9d;

Step 3. We kept moving the knife along the same direction and recorded the position of the knife (*x*_100-*F*_) when the total energy received by the detector reached (100-*F*)% of the total laser intensity, as shown in Figure 9e.

The laser radius can be calculated by [51]:

(3) $$\omega \left(z\right)=A\times \frac{\left|x_{F}-x_{100-F}\right|}{2}$$

where the value of *A* depends on *F*, which is shown in Table 2. When *F* equals 90, this method is also specified as 90/10 knife-edge method. Correspondingly, other methods can be referred to as 80/20, 70/30, and 60/40 methods.

The laser power intensity in the center of the laser beam *I*_0_ can be expressed as:

(4) $$I_{0}\left(z\right)=\frac{2\times Q}{\pi \omega^{2}\left(z\right)}$$

According to the measurement results of laser radius, when *L* = 20 cm, *ω* equals 1.116 mm. Therefore, *I*_0_ equals 51.1, 76.7, 89.5, and 102.2 mW/mm^2^, when *Q* equals 100, 150, 175, and 200 mW, respectively.

### 4.4. Experimental Setup

**Figure 10:**
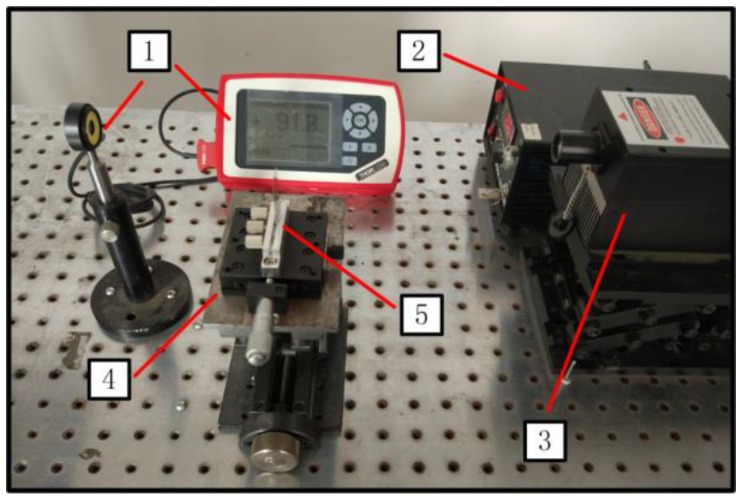
Laser radius measurement system. 1: photoelectric detector; 2: laser controller; 3: laser; 4: manual translation machine; 5: knife.

The experimental system using the knife-edge method to measure the radius of laser beam is shown in Figure 10. The laser used in the present research was purchased from Changchun New Industries Optoeletronics Technology Co., Ltd, and it is a diode laser at wavelength 1064 ± 1 nm (MIL-N-1064, Changchun China). The detector was purchased from Thorlabs Inc. (Shanghai, China) It has a handheld digital power meter (PM100D, Shanghai, China). and an energy meter (S120VC, Shanghai, China). The detectable wavelength range is 20–1100 nm. The measurement uncertainties are: ± 3% (440–980 nm); ± 5% (280–439 nm); ± 7% (200–279 nm, 981–1100 nm).

**Figure 11:**
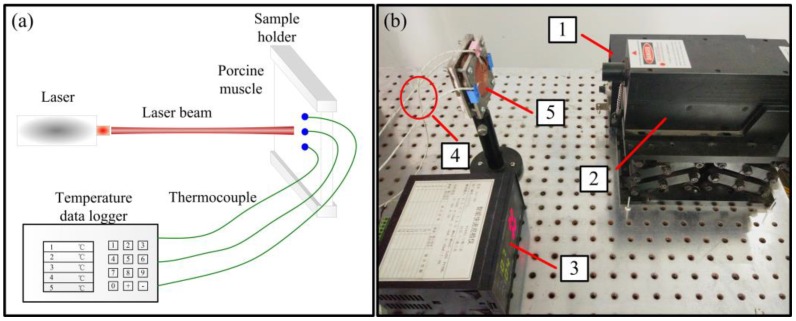
Laser heating experiment. (**a**) schematic diagram and (**b**) experimental system of interaction between laser and in vitro biological tissue. 1: laser controller; 2: laser; 3: temperature data logger; 4: thermocouple; 5: porcine muscle.

The measurement system of tissue thermal response is shown in Figure 11. The basic principle is to use a diode laser (1064 nm) to heat the porcine muscle. The time-dependent temperature response is measured by three thermocouples located around the laser spot.

## 5. Conclusions

In the present work, an experimental and theoretical investigation of photothermal conversion efficiency of GNTs and GNRs are presented. The DDA package was employed to study the spectral optical properties of GNRs and GNTs. The absorption quantum yield at resonance wavelengths were compared. Afterwards, the thermal effect of laser and in vitro tissue interaction was carried out. Also, a COMSOL model was applied to compare with the experimental results. To quantify the energy profile of Gaussian laser in COMSOL model, the laser radius was measured by knife-edge method. The main conclusions can be drawn as follows:

1. The overall photothermal conversion efficiency of GNTs is higher than that of GNRs in the commonly-used wavelengths for thermal therapy.

2. For GNRs, the localized surface plasmom resonance wavelength is highly dependent on the incident direction of electromagnetic wave. However, For GNTs, localized surface plasmom resonance wavelength in the long-wavelength zone is independent with the incident direction.

3. For GNRs loaded in in vitro tissue, high laser power and high concentration of nanoparticles will lead to the degeneration and even carbonization of tissue. For GNTs, the same situation (laser power, nanoparticle volume concentration, and heating time) could lead to the tissue’s evaporation instead of carbonization.

## Figures and Tables

**Table 1:** Value of *A* corresponding to different *F*. *L* = the distance between the measurement location and laser outlet. *A* and *F* are shown in Equation 3 in Materials and Methods.

*L*/cm	Measurement Position	Independent Measurement				
		1	2	3	4	5
20	*x*_90_ / mm	2.775	2.769	2.771	2.761	2.762
	*x*_10_ / mm	1.341	1.34	1.330	1.334	1.338
25	*x*_90_ / mm	2.628	2.638	2.639	2.632	2.637
	*x*_10_ / mm	1.220	1.223	1.226	1.222	1.223
27.5	*x*_90_ / mm	2.817	2.815	2.811	2.810	2.805
	*x*_10_ / mm	1.429	1.421	1.421	1.418	1.419
30	*x*_90_ / mm	2.825	2.825	2.86	2.866	2.863
	*x*_10_ / mm	1.978	1.976	2.029	2.031	2.032
32.5	*x*_90_ / mm	2.709	2.709	2.705	2.708	2.706
	*x*_10_ / mm	1.402	1.398	1.392	1.401	1.391

**Table 2:** Value of *A* corresponding to different *F*.

F	90	80	70	60
A	1.560	2.375	3.817	7.874

## References

[1] Huang X., El-Sayed M.A. (2011). Plasmonic photo-thermal therapy (PPTT). Alex. J. Med..

[2] Ren Y., Qi H., Chen Q., Ruan L. (2017). Thermal dosage investigation for optimal temperature distribution in gold nanoparticle enhanced photothermal therapy. Int. J. Heat Mass Transf..

[3] Singh R., Das K., Mishra S.C., Okajima J., Maruyama S. (2016). Minimizing tissue surface overheating using convective cooling during laser-induced thermal therapy: A numerical study. J. Therm. Sci. Eng. Appl..

[4] Mamalis A., Koo E., Sckisel G.D., Siegel D.M., Jagdeo J. (2016). Temperature-dependent impact of thermal aminolaevulinic acid photodynamic therapy on apoptosis and reactive oxygen species generation in human dermal fibroblasts. Br. J. Dermatol..

[5] Agah R., Gandjbakhche A.H., Motamedi M., Nossal R., Bonner R.F. (1996). Dynamics of temperature dependent optical properties of tissue: Dependence on thermally induced alteration. IEEE Trans. Biomed. Eng..

[6] Chen Q.W., Ying H.F., Gao S., Shen Y.H., Meng Z.Q., Chen H., Chen Z., Teng W.J. (2016). Radiofrequency ablation plus chemoembolization versus radiofrequency ablation alone for hepatocellular carcinoma: A systematic review and meta-analysis. Clin. Res. Hepatol. Gastroenterol..

[7] Wu F., Wang Z.B., Chen W.Z., Wang W., Gui Y., Zhang M., Zheng G., Zhou Y., Xu G., Li M. (2004). Extracorporeal high intensity focused ultrasound ablation in the treatment of 1038 patients with solid carcinomas in China: An overview. Ultrason. Sonochem..

[8] He N., Wang W., Ji Z., Li C., Huang B. (2010). Microwave ablation: An experimental comparative study on internally cooled antenna versus non-internally cooled antenna in liver models. Acad. Radiol..

[9] Peng X.H., Qian X., Mao H., Wang A.Y., Chen Z.G., Nie S., Shin D.M. (2008). Targeted magnetic iron oxide nanoparticles for tumor imaging and therapy. Int. J. Nanomed..

[10] Dombrovsky L.A., Timchenko V., Jackson M. (2012). Indirect heating strategy for laser induced hyperthermia: An advanced thermal model. Int. J. Heat Mass Transf..

[11] Dombrovsky L.A., Timchenko V., Jackson M., Yeoh G.H. (2011). A combined transient thermal model for laser hyperthermia of tumors with embedded gold nanoshells. Int. J. Heat Mass Transf..

[12] Huang X., Jain P.K., El-Sayed I.H., El-Sayed M.A. (2008). Plasmonic photothermal therapy (PPTT) using gold nanoparticles. Laser Med. Sci..

[13] Abadeer N.S., Murphy C.J. (2016). Recent progress in cancer thermal therapy using gold nanoparticles. J. Phys. Chem. C.

[14] Mackey M.A., Ali M.R.K., Austin L.A., Near R.D., El-Sayed M.A. (2014). The most effective gold nanorod size for plasmonic photothermal therapy: Theory and in vitro experiments. J. Phys. Chem. B.

[15] Soni S., Tyagi H., Taylor R.A., Kumar A. (2014). Investigation on nanoparticle distribution for thermal ablation of a tumour subjected to nanoparticle assisted thermal therapy. J. Therm. Biol..

[16] Krishnan S., Diagaradjane P., Cho S.H. (2010). Nanoparticle-mediated thermal therapy: Evolving strategies for prostate cancer therapy. Int. J. Hyperth..

[17] Elliott A.M., Shetty A.M., Wang J., Hazle J.D., Jason Stafford R. (2010). Use of gold nanoshells to constrain and enhance laser thermal therapy of metastatic liver tumours. Int. J. Hyperth..

[18] Huang X., El-Sayed M.A. (2010). Gold nanoparticles: Optical properties and implementations in cancer diagnosis and photothermal therapy. J. Adv. Res..

[19] Von Maltzahn G., Park J.H., Agrawal A., Bandaru N.K., Das S.K., Sailor M.J., Bhatia S.N. (2009). Computationally guided photothermal tumor therapy using long-circulating gold nanorod antennas. Cancer Res..

[20] Dickerson E.B., Dreaden E.C., Huang X., El-Sayed I.H., Chu H., Pushpanketh S., McDonald J.F., El-Sayed M.A. (2008). Gold nanorod assisted near-infrared plasmonic photothermal therapy (PPTT) of squamous cell carcinoma in mice. Cancer Lett..

[21] Huang X., Qian W., El-Sayed I.H., El-Sayed M.A. (2007). The potential use of the enhanced nonlinear properties of gold nanospheres in photothermal cancer therapy. Lasers Surg. Med..

[22] El-Sayed I.H., Huang X., El-Sayed M.A. (2006). Selective laser photo-thermal therapy of epithelial carcinoma using anti-EGFR antibody conjugated gold nanoparticles. Cancer Lett..

[23] Kennedy L.C., Bickford L.R., Lewinski N.A., Coughlin A.J., Hu Y., Day E.S., West J.L., Drezek R.A. (2011). A new era for cancer treatment: Gold-nanoparticle-mediated thermal therapies. Small.

[24] Jia Z., Cheng Q., Song J., Si M., Luo Z. (2016). Optical properties of a grating-nanorod assembly structure for solar cells. Opt. Commun..

[25] Anderson R.R., Parrish J.A. (1981). The optics of human skin. J. Investig. Dermatol..

[26] Huang X., Neretina S., El-Sayed M.A. (2009). Gold nanorods: From synthesis and properties to biological and biomedical applications. Adv. Mater..

[27] Mallick S., Sun I.C., Kim K., Yil D.K. (2013). Silica coated gold nanorods for imaging and photo-thermal therapy of cancer cells. J. Nanosci. Nanotechnol..

[28] Tarapacki C., Kumaradas C., Karshafian R. (2013). Enhancing laser thermal-therapy using ultrasound-microbubbles and gold nanorods of in vitro cells. Ultrasonics.

[29] Grześkiewicz B., Ptaszyński K., Kotkowiak M. (2014). Near and far-field properties of nanoprisms with rounded edges. Plasmonics.

[30] Jin R., Cao Y., Mirkin C.A., Kelly K.L., Schatz G.C., Zheng J.G. (2001). Photoinduced conversion of silver nanospheres to nanoprisms. Science.

[31] Ren Y., Qi H., Chen Q., Wang S., Ruan L. (2017). Localized surface plasmon resonance of nanotriangle dimers at different relative positions. J. Quant. Spectrosc. Radiat. Transf..

[32] Bazán-Díaz L., Mendoza-Cruz R., Velázquez-Salazar J.J., Plascencia-Villa G., Romeu D., Reyes-Gasga J., Herrera-Becerra R., José-Yacamán M., Guisbiers G. (2015). Gold-copper nanostars as photo-thermal agents: Synthesis and advanced electron microscopy characterization. Nanoscale.

[33] Pang B., Yang X., Xia Y. (2016). Putting gold nanocages to work for optical imaging, controlled release and cancer theranostics. Nanomedicine.

[34] Chen J., Glaus C., Laforest R., Zhang Q., Yang M., Gidding M., Welch M.J., Xia Y. (2010). Gold nanocages as photothermal transducers for cancer treatment. Small.

[35] Xie J., Zhang Q., Lee J.Y., Wang D.I. (2008). The synthesis of SERS-active gold nanoflower tags for in vivo applications. ACS Nano.

[36] Rai A., Singh A., Ahmad A., Sastry M. (2006). Role of halide ions and temperature on the morphology of biologically synthesized gold nanotriangles. Langmuir.

[37] Luo L., Nie C., Du P., Hongwei Z., Wei W., Zhang M., Ambati B., Sun Z. (2014). An Efficient Near-Infrared Photothermal Therapy Agent by Using Ag@ Oxides Nanoprisms in for Uveal melanoma Therapy. Investig. Ophthalmol. Vis. Sci..

[38] Bao C., Conde J., Pan F., Li C., Zhang C., Tian F., Liang S., Fuente J.M.D.L., Cui D. (2016). Gold nanoprisms as a hybrid in vivo cancer theranostic platform for in situ photoacoustic imaging, angiography, and localized hyperthermia. Nano Res..

[39] Hodkinson J.R., Greenleaves I. (1963). Computations of light-scattering and extinction by spheres according to diffraction and geometrical optics and some comparisons with the Mie theory. J. Opt. Soc. Am..

[40] Flatau P.J., Draine B.T. (2012). Fast near field calculations in the discrete dipole approximation for regular rectilinear grids. Opt. Express.

[41] Johnson P.B. (1972). Optical constants of the noble metals. Phys. Rev. B.

[42] Zhang B., Xu C.L., Wang S.M. (2016). An inverse method for flue gas shielded metal surface temperature measurement based on infrared radiation. Meas. Sci. Technol..

[43] Ren Y., Qi H., Zhao F., Ruan L., Tan H. (2016). Simultaneous retrieval of temperature-dependent absorption coefficient and conductivity of participating media. Sci. Rep..

[44] Liu K., Xue X., Furlani E.P. (2016). Theoretical Comparison of Optical Properties of Near-Infrared Colloidal Plasmonic Nanoparticles. Sci. Rep..

[45] Niemz M.H. (2007). Laser-Tissue Interactions.

[46] Draine B.T., Flatau P.J. (1994). Discrete-dipole approximation for scattering calculations. J. Opt. Soc. Am. A.

[47] Lee K.-S., El-Sayed M.A. (2005). Dependence of the enhanced optical scattering efficiency relative to that of absorption for gold metal nanorods on aspect ratio, size, end-cap shape, and medium refractive index. J. Phys. Chem. B.

[48] Draine B.T. (1988). Discrete-dipole approximation and its application to interstellar graphite grains. Astrophys. J..

[49] Flatau P.J. (1997). Improvements inthe discrete dipole approximation method of computing scattering and absorption. Opt. Lett..

[50] De Araújo M.A., Silva R., De L.E., Pereira D.P., de Oliveira P.C. (2009). Measurement of Gaussian laser beam radius using the knife-edge technique: Improvement on data analysis. Appl. Opt..

[51] Fan X., Zheng Y., Sun Q., Wang G., Zeng H., Bing P., Ren H. (2008). Experimental study on measuring the beam waist of Gaussian laser beam using 90/10 knife edge method. Laser Infrared.

[52] Khosrofian J.M., Garetz B.A. (1983). Measurement of a Gaussian laser beam diameter through the direct inversion of knife-edge data. Appl. Opt..

[53] Yoshida A., Asakura T. (1976). A simple technique for quickly measuring the spot size of Gaussian laser beams. Opt. Laser Technol..

